# Supplementary material for: Non-selective distribution of infectious disease prevention may outperform risk-based targeting
Source: Nat Commun. 2022 May 31;13:3028. doi: 10.1038/s41467-022-30639-3 (PMC9156732; doi:10.1038/s41467-022-30639-3)
Supplement: Supplementary file 1 — Supplementary Information [file 41467_2022_30639_MOESM1_ESM.pdf]

Supplementary Information for

Non-selective distribution of infectious disease prevention may outperform risk-based targeting

Benjamin Steinegger[1], Iacopo Iacopini[2,3], Andreia Sofia Teixeira[4,5], Alberto Bracci[6], Pau Casanova-Ferrer[7,8], Alberto Antonioni[7], and Eugenio Valdano[9,*].

[1]Departament d'Enginyeria Informàtica i Matemàtiques, Universitat Rovira i Virgili, 43007 Tarragona, Spain.
[2]Department of Network and Data Science, Central European University, 1100 Vienna, Austria.
[3]Aix Marseille Univ, Université de Toulon, CNRS, CPT, Marseille, 13009, France.
[4]LASIGE, Departamento de Informática, Faculdade de Ciências, Universidade de Lisboa, 1749–016 Lisboa, Portugal.
[5]INESC-ID, Lisboa, Portugal
[6]Department of Mathematics, City, University of London, EC1V 0HB London, UK.
[7]Grupo Interdisciplinar de Sistemas Complejos (GISC), Department of Mathematics, Carlos III University of Madrid, Leganés, Spain.
[8]Department of Systems Biology, Centro Nacional de Biotecnología, CNB-CSIC, 28049 Madrid, Spain.
[9]Sorbonne Université, INSERM, Institut Pierre Louis d'Epidémiologie et de Santé Publique, F75012, Paris, France

[*] Corresponding author eugenio.valdano@inserm.fr

Supplementary Note 1 The impact of degree-assortativity on the epidemic dynamics

In the the main text we assumed that individuals mixed randomly according to their degree, i.e., there were no degree-degree correlations along contacts. We treat here the case in which individuals mix preferably within the same activity class (degree), which is especially relevant in the case of sexual encounters.[1] This property is formally referred to as degree-assortativity.[2] We extended the model to test whether the latter affects the conclusions drawn in the main text. We incorporated degree-assortativity according to the approach of Refs.[3,4] In this case, the differential equations describing the epidemic evolution (compare with Eq. (1) of the main text) read

$$\begin{cases} \dot{x}_k = -\mu x_k + \lambda k(1-x_k)\xi_k \\ \dot{y}_k = -\mu y_k + \lambda(1-\epsilon)k(1-y_k)\xi_k \\ \xi_k = \sum\limits_m \mathrm{L}_{km}\left[(1-g_m)x_m + g_m y_m\right] \\ \mathrm{L}_{km} = \left[\omega\delta_{km} + (1-\omega)\frac{m p_m}{\langle k\rangle}\right] . \end{cases} \tag{S1}$$

Within this framework, degree-assortativity is encoded in the contact structure L_{km} and controlled through the assortativity parameter $\omega \in [0,1]$. Specifically, the probability that a node with degree k has a contact with a node with degree m is a mixture distribution between the probability distribution of random mixing ($mp_m/\langle k\rangle$),[5] and full degree assortativity ($k = m$). ω then tunes assortativity, by changing the weights of the distributions in the mixture. In the special case of $\omega = 0$ (no assortativity), then L_{km} no longer depends on k, which implies that $\xi_k = \xi$ no longer depends on k, and causes Eq. (S1) to correctly coincide with Eq. (1) of the main paper. Also, given that $p(m)$ decreases with m, the excess probability of assortative mixing given a certain value of ω increases as degree increases, correctly modeling high-risk individuals mixing preferably with high-risk individuals.

Supplementary Note 1.1 Calculating the response function $f(k)$

Given this set of equations, we proceed to calculate $f(k)$ in a way that is formally similar to what we did in the main paper. Accordingly, we assume equilibrium ($\dot{x} = 0$), and take the derivative of the first line in Eq. (S1) leading to

$$-\mathrm{J}_{km} + \frac{\lambda}{\mu}k\left[-\mathrm{J}_{km}\xi_k + (1-x_k)\frac{d\xi_k}{dg_m}\right] = 0\,. \tag{S2}$$

Calculating the derivative $d\xi_k/dg_m$, we find

$$\frac{d\xi_k}{dg_m} = \mathrm{L}_{km}(y_m - x_m) + \sum_h \mathrm{L}_{kh}\mathrm{J}_{hm}\,. \tag{S3}$$

Inserting Eq. (S3) into Eq. (S2), we get

$$\sum_h \left[\left(1 + \frac{\lambda}{\mu}k\left(\frac{z(1-\omega)}{\langle k\rangle} + \omega x_k\right)\right)\delta_{kh} - \frac{\lambda}{\mu}k(1-x_k)\mathrm{L}_{kh}\right]\mathrm{J}_{hm} = \frac{\lambda}{\mu}k(1-x_k)\mathrm{L}_{km}(y_m - x_m)\,. \tag{S4}$$

The above equation can be written in a more compact way by defining the following quantities

$$\begin{cases} \mathrm{d}_k = 1 + \frac{\lambda}{\mu}k\left[\frac{z}{\langle k\rangle} + \omega\left(2x_k - 1 - \frac{z}{\langle k\rangle}\right)\right] \\ \mathrm{D}_{ij} = \mathrm{d}_i\delta_{ij} \\ \mathrm{u}_k = -\frac{\lambda(1-\omega)}{\mu\langle k\rangle}k(1-x_k) \\ \mathrm{v}_k = kp_k \\ \mathrm{A}_{km} = \frac{\lambda}{\mu}k\mathrm{L}_{km}(y_m - x_m)\,. \end{cases} \tag{S5}$$

More specifically, Eq. (S4) becomes

$$\left(\mathbf{D} + \mathbf{u}\mathbf{v}^T\right)\mathbf{J} = \mathbf{A}\,. \tag{S6}$$

Therefore we can invert the above expression taking advantage of the Sherman-Morrison formula[6] leading to

$$\mathbf{J} = \left(\mathbf{D}^{-1} - \frac{\mathbf{D}^{-1}\mathbf{u}\mathbf{v}^T\mathbf{D}^{-1}}{1 + \mathbf{v}^T\mathbf{D}^{-1}\mathbf{u}}\right)\mathbf{A}\,. \tag{S7}$$

The above expression of $\mathrm{J}_{km} = dx_k/dg_m$ can then be inserted into the expression of $f(k)$ in Eq. (2) giving us

$$f(k) = (x_k - y_k)\left[1 + \frac{\lambda}{\mu}k\left((1-\omega)\Gamma + \omega\frac{(1-x_k)(1+ck)}{\mathrm{d}_k}\right)\right] \tag{S8}$$

where we defined

$$\begin{cases} c = -\frac{\sum_k p_k \frac{\mathrm{u}_k}{\mathrm{d}_k}}{1 + \sum_k \frac{\mathrm{u}_k \mathrm{v}_k}{\mathrm{d}_k}} \\ \Gamma = \frac{1}{\langle k\rangle}\sum_k p_k \frac{k(1-x_k)(1+ck)}{\mathrm{d}_k}\,. \end{cases} \tag{S9}$$

From Eq. (S8) we can directly identifiy $F_{dir}(k)$ and $F_{indir}(k)$:

$$F_{dir}(k) = x_k - y_k \tag{S10}$$

$$F_{indir}(k) = (x_k - y_k)\,\frac{\lambda}{\mu}k\left((1-\omega)\Gamma + \omega\frac{(1-x_k)(1+ck)}{\mathrm{d}_k}\right)n\,. \tag{S11}$$

The variables y_k can be expressed as a function of x_k through Eq. (9) of the main paper, which also holds in the presence of assortativity. Furthermore, after some algebra, the equilibrium condition $\dot{x_k} = 0$ is found as

$$\frac{\lambda}{\mu}\omega k x_k^2 + \left[1 + \frac{\lambda}{\mu}(1-\omega)k\frac{z}{\langle k \rangle} - \frac{\lambda}{\mu}\omega k\right] x_k - \frac{\lambda}{\mu}k(1-\omega)\frac{z}{\langle k \rangle} = 0\,. \qquad \text{(S12)}$$

The above equation is the equivalent of Eq. (10) in the main text. However, here the equation is not linear but of second degree, and inserting an analytical expression of x_k into Eq. S8 is no longer feasible. For this reason, we evaluated Eq. (S8) and (S12) numerically.

Supplementary Note 1.2 The dependence of $f(k)$ on assortativity

Figure S1 shows the $F_{dir}(k)$ and $F_{indir}(k)$ for different values of ω (assortativity parameters). Assortativity has little effect on $F_{dir}(k)$. Its impact on $F_{indir}(k)$ is instead more pronounced. By increasing the likelihood of contact among high-degree nodes, assortativity has the effect of increasing risk of exposure among those who are already at high risk of exposure (high degree). As such, it increases the probability of breakthrough infections among high-risk nodes. This further skews the tradeoff between protecting high-risk nodes, and avoiding breakthrough infections, toward the latter aspect. In practical terms, assuming all other parameters are kept constant, increasing assortativity means enhancing the phenomenology presented in the main paper. Specifically, Fig. S1 shows that k^* continuously decreases with increasing assortativity. Furthermore, the decrease in $f(k)$ as k increases is sharper in the presence of assortativity. The same can be observed in Fig. S1c, which shows $f(k)$ for different values of ϵ, with and without degree-assortativity. Most importantly, Fig. S1c shows that assortativity can cause k^* to exist, even when $f(k)$ would be monotonously increasing in the case of no assortativity. This means that assortativity increases the value of the critical transmissibility ϵ_c, as the next section will explain.

Supplementary Note 1.3 The dependence of ϵ_c and ϵ_r on assortativity

We studied the impact of assortativity on ϵ_c, ϵ_r, and the implications on the estimated optimal PrEP distribution in MSM communities.

Ref.[1] estimates $\omega = 0.14$ for contacts among MSM in Sweden. In the following we tested this value ($\omega = 0.14$), and twice this value ($\omega = 0.28$). The latter is a very high value of assortativity: with the parameters as in Fig. S1b, those with $k \geq 5$ would come into contact with others with $k \geq 5$ roughly one third of the time: compare this with only 1/20th of the time in the case of no assortativity. We included such high value of assortativity to explore communities with mixing patterns which might be very different from the

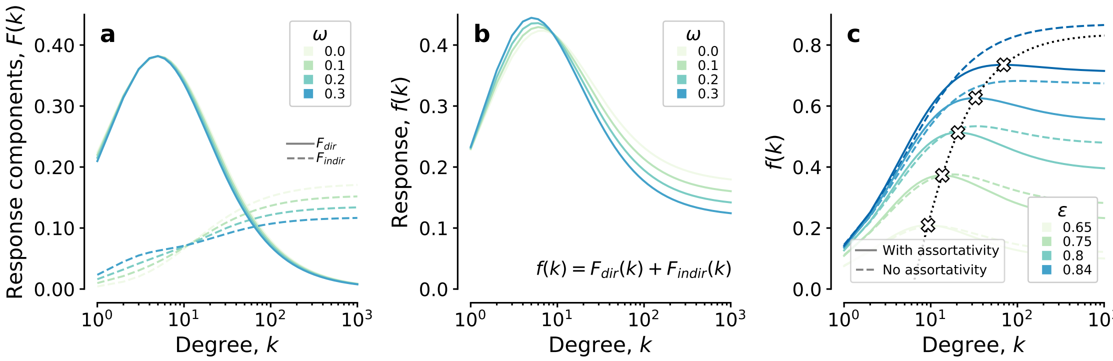

Figure S1: **(a)** Terms $F_{dir}(k)$ (Eq. (S10)) and $F_{indir}(k)$ (Eq. (S11)) for different values of assortativity, ω. **(b)** Response function $f(k)$, which is the sum of the terms in (a). **(c)** Response function $f(k)$ for different values of ϵ with $\omega = 0.14$ (solid lines) and with $\omega = 0$ (dashed lines). The black dashed line and crosses indicates k^* in the presence of assortativity. The other parameters are the same as in Fig. 1 of the main paper: Reduced transmissibility is $\hat{\lambda} = 2$; degree distribution is a negative binomial with mean 2.0, coefficient of variation 4.7.

Swedish study, which can be hardly regarded as representative of different socioeconomic and epidemiological contexts.

Fig. S2a shows that assortativity increases both ϵ_c and ϵ_r. Notwithstanding, Fig. S2b shows that, for $\omega = 0.14$, only four communities moved from from the high-efficacy region to the transition zone, out of the 34 assigned to the high-efficacy region in the case of no assortativity (main paper). Plus, no communities in the low-efficacy region and transition zone changed region assignment due to assortativity. Fig. S2c shows region assignments in the case of $\omega = 0.28$: In this case, compared to the analysis in the main text, 7 communities moved from the high-efficacy region to the transition zone, and 2 communities moved from the transition zone to the low-efficacy zone.

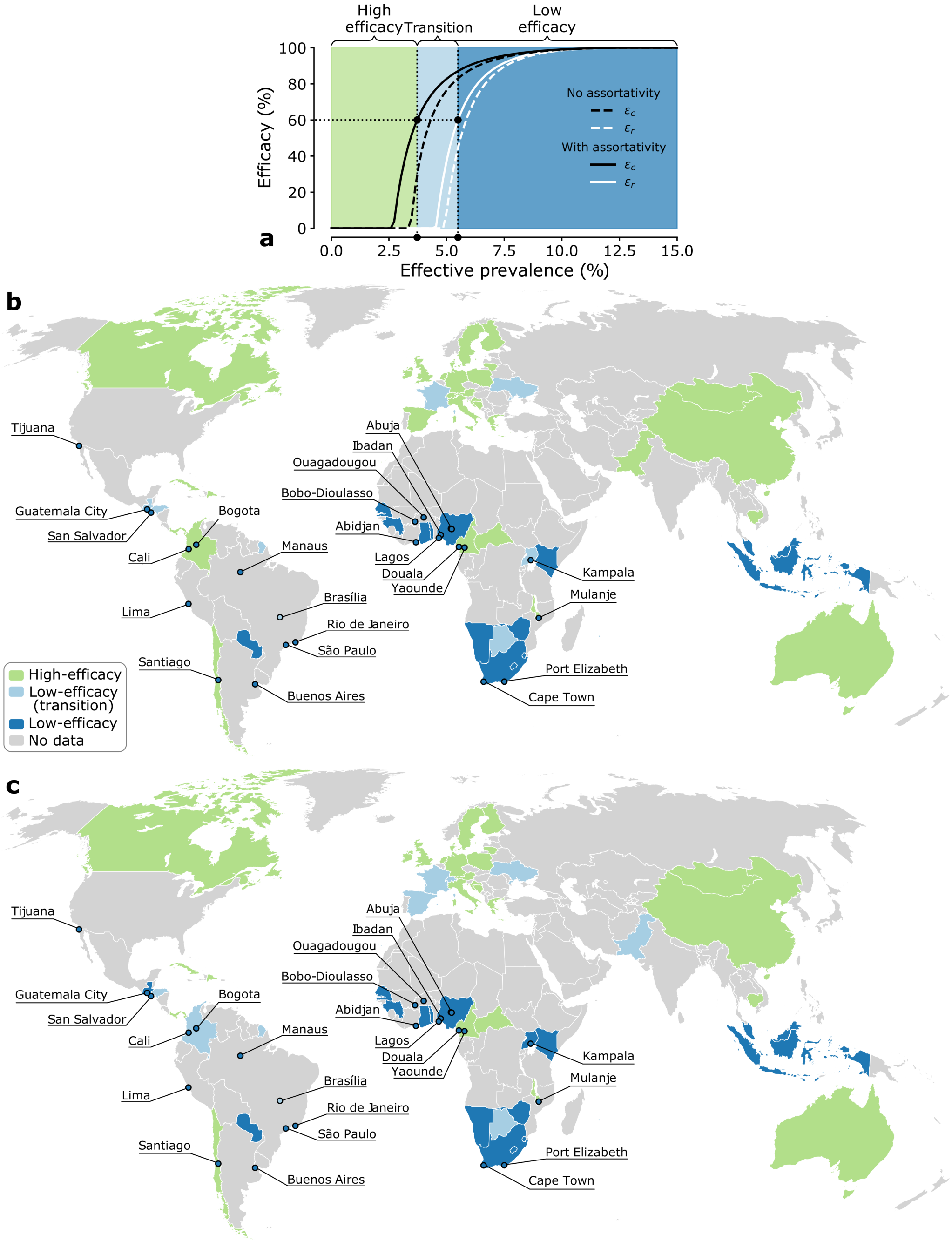

Figure S2: **(a)** Phase diagram of PrEP in MSM communities. The x-axis shows the effective prevalence, i.e., the fraction of individuals who are living with HIV and can potentially transmit it. The y-axis shows efficacy of PrEP. The horizontal dashed line is 60% efficacy. The black curve is critical efficacy ϵ_c, the white curve is ϵ_r. While the solid lines report the results in the presence of assortativity ($\omega = 0.14$), the dashed lines report the results of the main paper (no assortativity, $\omega = 0.0$). The range of effective prevalence in the high-efficacy region at 60% efficacy is colored in green. The range of effective prevalence in the low-efficacy region at 60% efficacy is colored in dark blue, and light blue (transition zone). **(b)** & **(c)** Maps showing parameter region estimates in 58 countries, 24 cities with assortativity fixed as $\omega = 0.14$ (b) and $\omega = 0.28$ (c), respectively. Communities in the high-efficacy region are green, communities in the low-efficacy region are in dark blue, light blue (transition zone).

Supplementary Note 2 Timescale separation

Annealed networks are reliable when contacts change much faster than the characteristic time scale of disease spread. We prove here that this is true in the case of HIV epidemics in MSM communities. We focus on the type of links in the network that change at the fastest pace: one-time partnerships, which, according to Ref.,[7] have a mean rate of acquisition $r_{network} = 0.16\, week^{-1}$. Thus, the lower bound of the time scale of network evolution is $\tau_{network} \approx 6\, week$. Then, we focus on the contact at highest risk of HIV acquisition: unprotected, receptive anal intercourse, which has a per-act probability of transmission of $p = 0.014$ (Ref.[8]). Thus, the lower bound of the time scale of disease transmission is $\tau_{disease} = (p\, r_{network})^{-1} \approx 450 \gg \tau_{network}$. This estimate of $\tau_{disease}$ is indeed a lower bound, because it assumes that all acts are highest-risk acts, and that all partners may spread HIV: the actual disease is thus probably much higher. This proves the time scale separation.

Supplementary Note 3 Population turnover and treatment

Equation (1) of the main paper represents a Susceptible-Infected-Susceptible model, which is a simple representation of HIV dynamics by effectively using the "recovery" parameter μ as a replacement mechanism. We show here rigorously that, despite its simplicity, this model effectively includes replacement dynamics in the community, and treatment.

Let us write new equations, which consider all transitions (and in particular treatment and population turnover) explicitly:

$$\begin{cases} \dot{X}_k = -\mu^{(i)} X_k + \frac{\lambda k}{\langle k \rangle} S_k^{(x)} \Xi - \rho X_k \\ \dot{Y}_k = -\mu^{(i)} Y_k + \frac{\lambda k)}{\langle k \rangle} S_k^{(y)} \Xi - \rho Y_k \\ \dot{S}_k^{(x)} = -\frac{\lambda k}{\langle k \rangle} S_k^{(x)} \Xi + \sigma_k - \mu^{(s)} S_k^{(x)} - \nu_k \\ \dot{S}_k^{(y)} = -\frac{\lambda(1-\epsilon)k}{\langle k \rangle} S_k^{(y)} \Xi - \mu^{(s)} S_k^{(y)} + \nu_k \\ \dot{R}_k = \rho(X_k + Y_k) - \omega_k \\ \Xi = \frac{1}{N} \sum_m m \left[X_m + Y_m \right] \end{cases} \tag{S13}$$

The variables here are numbers of individuals, not fractions. $S_k^{(x)}$ is the number of individuals with k contacts, not on PrEP, who are susceptible. X_k is the number of individuals with k contacts, not on PrEP, who are susceptible. $S_k^{(y)}, Y_k$ are defined analogously. R_k is the number of infected individuals with k contacts who are on treatment and cannot transmit (virally suppressed). The total population is $N = \sum_k (X_k + Y_k + S_k^{(x)} + S_k^{(y)} + R_k)$, and, without additional constraints, can vary in time (we will impose the constraint of fixed population later). $\mu^{(i)}$ is the rate at which an infected individual leaves community for any reason; ρ is the rate at which an infected individual becomes virally suppressed thanks to treatment. σ_k is the term accounting for new individuals joining the community. This assumes that new individuals are susceptible and not on PrEP, which is compatible with them being young individuals becoming sexually active. The term ν_k encodes the rate of PrEP adoption. Finally, the term ω_k encodes treated individuals leaving the community.

We will now prove that the dynamics described by Eq. (1) of the main paper is equivalent to Eq. (S13), provided that the three following conditions hold: i) the population of the community is constant in time; ii) PrEP coverage is constant in time; iii) treatment is

constant in time. These conditions translate as follows:

$$\begin{cases} -\mu^{(i)}(X_k + Y_k) - \mu^{(s)}(S^{(x)} + S^{(y))} + \sigma_k - \omega_k = 0 \\ \rho(X_k + Y_k) - \omega_k = -g_k^{-1}\left[-(\mu^{(i)} + \rho)Y_k - \mu^{(s)}S_k^{(y)} + \nu_k\right] \\ \rho(X_k + Y_k) - \omega_k = 0 \end{cases} \tag{S14}$$

which give the expressions of the generic terms $\sigma_k, \nu_k, \omega_k$:

$$\sigma_k = (\mu^{(i)} + \rho)(X_k + Y_k) + \mu^{(s)}(S_k^{(x)} + S_k^{(y)}) \tag{S15}$$

$$\nu_k = (\mu^{(i)} + \rho)Y_k + \mu^{(s)}S_k^{(y)} \tag{S16}$$

$$\omega_k = \rho(X_k + Y_k) \tag{S17}$$

Inserting these into Eq. (S13), one gets

$$\begin{cases} \dot{X}_k = -\mu X_k + \frac{\lambda k}{\langle k \rangle} S_k^{(x)} \Xi \\ \dot{Y}_k = -\mu Y_k + \frac{\lambda k)}{\langle k \rangle} S_k^{(y)} \Xi \\ \dot{S}_k^{(x)} = -\frac{\lambda k}{\langle k \rangle} S_k^{(x)} \Xi + \mu X_k \\ \dot{S}_k^{(y)} = -\frac{\lambda(1-\epsilon)k}{\langle k \rangle} S_k^{(y)} \Xi + \mu Y_k \\ \dot{R}_k = 0 \\ \Xi = \frac{1}{N}\sum_m m\left[X_m + Y_m\right] \end{cases} \tag{S18}$$

where $\mu = \mu^{(i)} + \rho$. We already see that we are almost arrived at the model of the main paper: Equation (S18) has become that of an SIS model, where both $X_k + S_k^{(x)}$, and $Y_k + S_k^{(y)}$ are conserved in time, and a fixed number of individuals (R) is infected but cannot transmit. Lines 3-5 in Eq. (S18) are thus now redundant. We now recover variables x_k, y_k of the main paper as follows:

$$x_k = \frac{X_k}{(N_k - R_k)(1 - g_k)} = \frac{1}{N}\frac{X_k}{p_k(1 - r_k)(1 - g_k)}; \tag{S19}$$

$$y_k = \frac{Y_k}{(N_k - R_k)g_k} = \frac{1}{N}\frac{Y_k}{p_k(1 - r_k)g_k}, \tag{S20}$$

where $r_k = R_k/N_k$. We then divide lines 1 in Eq. (S18) by $(N_k - R_k)(1 - g_k)$, and line 2 by $(N_k - R_k)g_k$. Finally, we assume that the probability of being on treatment does not depend on contacts ($r_k = r$), and rewrite Ξ in terms of x_k, y_k, getting $\Xi = (1 - r)\xi$ (ξ of the main paper).

With that, we proved that Eq. (S13) becomes Eq. (1) of the main paper, under the above assumptions. In particular, we proved that

- the replacement rate μ effectively models both population turnover (new people coming in the community, and people going out of the community), and treatment initiation;
- the presence of treatment effectively rescales transmissibility $\lambda \longrightarrow (1-r)\lambda$, where r is treatment coverage.

Supplementary Note 4 Scale-free distribution of the contact network

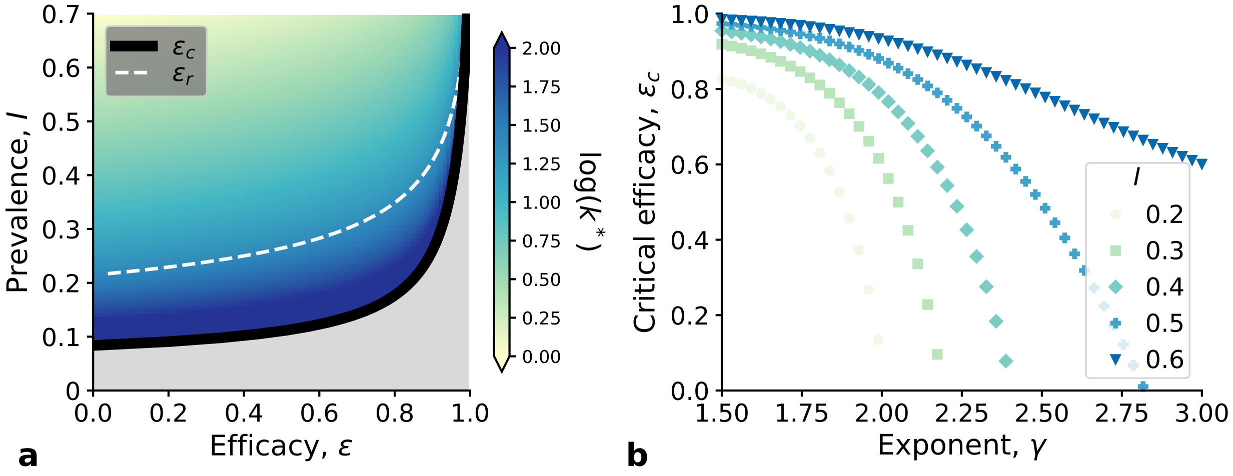

Figure S3: Same as Fig. 2 in the main text but for a contact network that follows a scale-free distribution, i.e. $p(k) \sim k^{-\gamma}$. In panel (a) we fix $\gamma = 1.5$. The phenomenology is qualitatively the same as what observed in the main paper. Specifically, in (b), critical efficacy is higher when heterogeneity in the contact network is higher (lower exponents).

Supplementary Note 5 Numerical evaluation of $f(k)$, $F_{dir}(k)$ and $F_{indir}(k)$

The mathematical analysis enabled us to get expression for the linear response function $f(k)$. We explained the observed phenomenology through the calculations $F_{dir}(k)$ and $F_{indir}(k)$. To verify that the mathematical analysis was correctly performed, we calculated numerically the values of the functions $f(k)$, $F_{dir}(k)$ and $F_{indir}(k)$. For the function $F_{dir}(k)$ this means to solve numerically Eq. (1) of the main text and evaluating $y_k - x_k$. To calculate $F_{indir}(k)$, we numerically evaluate the derivative dx_m/dg_k. We do so by introducing a small fraction of individuals on PrEP in degree class k, solve the equations in Eq. (1), calculate the derivative through finite differences and repeat these steps for all the degree classes. For evaluating $f(k)$ we take the same approach, but evaluate the derivative with respect to the overall prevalence. Fig. S4 shows the that the numerical evaluation of $f(k)$, $F_{dir}(k)$ and $F_{indir}(k)$ matches perfectly the analytical expressions.

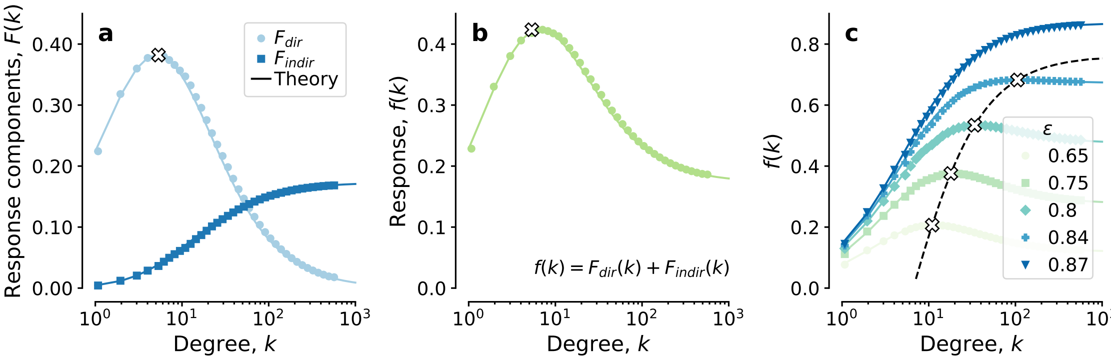

Figure S4: Same as Fig. 1 in the main text but here we compare the numerical results (points) with the analytical curves (continuous lines) presented in the main text.

Supplementary Note 6 HIV epidemiological data and estimates

Tables S1, S2, S3, S4 show the sources of the estimates of HIV prevalence, ART (antiretroviral treatment) coverage, viral suppression, used to compute ϵ_c, ϵ_r across communities of men-having-sex-with-men (MSM) (see Fig. 3 of the main paper).

When Ref.[9] was the source, we used ART coverage and viral suppression estimates among MSM when available, among the general male population otherwise.

In Latin America, we used country-specific ART coverage and viral suppression rate.[10] When no viral suppression data where available, we assumed 50 % viral suppression rate among those on ART, as consistent with Ref.[10]

In Africa, when specific ART coverage and viral suppression was not available, we used the averages from the meta-analysis at Ref.[11]

In each MSM community considered, we computed effective prevalence as the probability that a person is living with HIV, and is not virally suppressed. If I is HIV prevalence, a is ART coverage, v is viral suppression rate among those on ART, then the effective prevalence is $I_{eff} = (1 - av)I$. Tables S1, S2, S3, S4 report the values of effective prevalence.

We describe the distribution of sexual contacts with a negative binomial. We fix the average degree and standard deviation according to Ref.[12] as 2.1 and 11.6. The study reports the number of partners with which individuals had unprotected anal intercourse in the past year in the UK. Data was collected from 2000 until 2013.

Table S1: **Europe**

	Location	Effective Prevalence	Source
1	Denmark	1.2E-03	[13,14]
2	Finland	1.5E-03	[9]
3	Croatia	2.5E-03	[13,14]
4	Sweden	3.1E-03	[13,14]
5	Bosnia and Herzegovina	6.3E-03	[9]
6	Italy	8.1E-03	[13,14]
7	Austria	1.0E-02	[13,14]
8	Greece	1.0E-02	[13,14]
9	Germany	1.2E-02	[9]
10	United Kingdom	1.7E-02	[9]
11	Ireland	1.8E-02	[9]
12	Lithuania	1.9E-02	[9]
13	Netherlands	2.3E-02	[9]
14	Poland	2.8E-02	[9]
15	Switzerland	3.3E-02	[9]
16	Spain	3.5E-02	[9]
17	France	3.7E-02	[9]
18	Ukraine	4.3E-02	[9]

Table S2: **South and Central America:**

	Location	Effective Prevalence	Source
1	Cuba	4.9E-03	[9]
2	Panama	1.1E-02	[9]
3	Barbados	1.9E-02	[9]
4	Chile	2.8E-02	[9]
5	Dominican Republic	2.8E-02	[9]
6	Colombia	3.5E-02	[9]
7	Honduras	4.6E-02	[9]
8	Brasilia	4.6E-02	[10,15]
9	Guatemala	5.2E-02	[9]
10	San Salvador	9.4E-02	[10,15]
11	Guatemala City	1.0E-01	[10,15]
12	Manaus	1.2E-01	[10,15]
13	Rio de Janeiro	1.2E-01	[10,15]
14	Buenos Aires	1.3E-01	[10,15]
15	Santiago de Chile	1.3E-01	[10,15]
16	Bogota	1.4E-01	[10,15]
17	Lima	1.7E-01	[10,15]
18	Paraguay	1.7E-01	[9]
19	Sao Paulo	1.8E-01	[10,15]
20	Tijuana	2.0E-01	[10,15]
21	Cali	2.0E-01	[10,15]

Table S3: **North America, Asia and Australia**

	Location	Effective Prevalence	Source
1	Cambodia	2.9E-03	[9]
2	China	7.8E-03	[9]
3	Canada	1.3E-02	[9]
4	Australia	2.0E-02	[9]
5	Pakistan	3.7E-02	[9]
6	Malaysia	1.0E-01	[9]
7	Indonesia	1.2E-01	[9]

Table S4: **Africa**

	Location	Effective Prevalence	Source
1	Malawi	2.4E-02	[9]
2	Cameroon	2.6E-02	[9]
3	Central African Republic	3.0E-02	[9]
4	Botswana	4.1E-02	[9]
5	Uganda	5.4E-02	[9]
6	South Africa	6.7E-02	[9]
7	Kenya	7.5E-02	[9]
8	Guinea	7.5E-02	[9]
9	Zimbabwe	7.5E-02	[9]
10	Ghambia	8.0E-02	[11,16]
11	Togo	9.8E-02	[9]
12	Namibia	1.0E-01	[11,17]
13	Kampala (Uganda)	1.2E-01	[11,18,19]
14	Ougadougou	1.2E-01	[11,20,21]
15	Swaziland	1.4E-01	[11,17]
16	Abidjan (Côte d'Ivoire)	1.4E-01	[11,22]
17	Nigeria	1.6E-01	[9]
18	Ghana	1.8E-01	[9]
19	Senegal	1.9E-01	[9]
20	Bobo Dioulasso (Burkina Faso)	1.9E-01	[11,20]
21	Abuja (Nigeria)	2.2E-01	[11,23]
22	Ibadan (Nigeria)	2.3E-01	[11,23]
23	Mulanje (Malawi)	2.3E-01	[11,24]
24	Douala (Cameroon)	2.3E-01	[11,25]
25	Cape Town	2.4E-01	[11,26,27]
26	Lesotho	2.7E-01	[11,28]
27	Gambia	3.3E-01	[9]
28	Lagos (Nigeria)	3.6E-01	[11,23]
29	Youndé (Cameroon)	4.0E-01	[11,25]
30	Port Elizabeth	4.1E-01	[11,26,27]

Supplementary Note 7 Phase assignment vs. treatment coverage

The table S5 shows the number of cities and countries in the two distinct phases – low-efficacy, high-efficacy – inside four different groups of antiretroviral treatment (ART) coverage. High ART coverage is generally associated with the high-efficacy phase. Low ART coverage is generally associated with the low-efficacy phase.

Table S5: **Association between phase and treatment coverage**

ART coverage	low-efficacy	low-efficacy (transition zone)	high-efficacy
0-25%	21	0	1
25-50%	12	2	4
50-75%	7	1	5
75-100%	0	1	24

Supplementary Note 8 COVID-19 vaccination

To examine the possible existence of a low-efficacy phase for COVID-19 vaccination, we used a negative-binomial degree distribution with mean 13.4,[29] coefficient of variation ranging from 0.27 (Poisson distribution), to 3.17.[30] We fixed the infectious period to $\mu^{-1} = 7.5$ days.[31] We then computed ϵ_c at varying levels of weekly incidence. Fig. S5 shows that the low-efficacy phase exists only at unrealistically high weekly incidence, even in the most heterogeneous case, in terms of contact network.

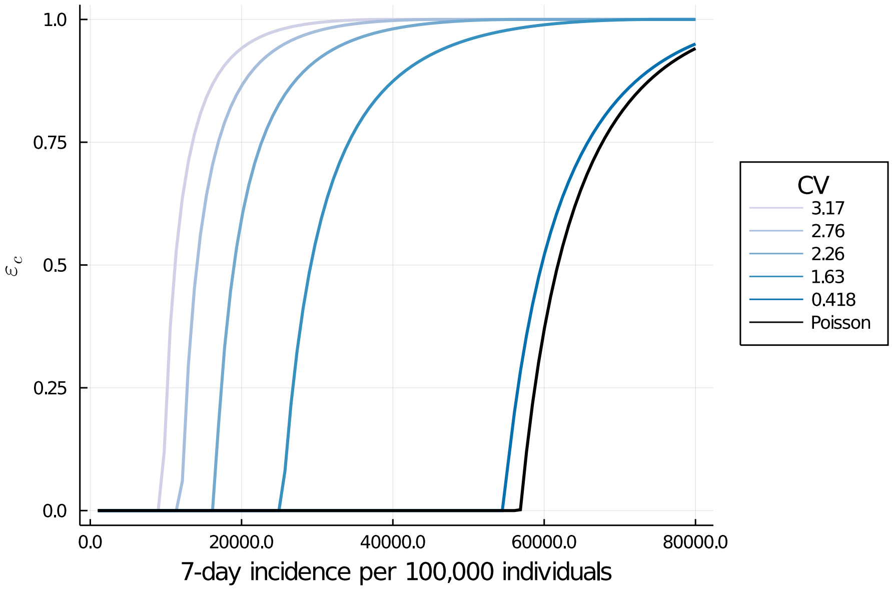

Figure S5: Dependence of ϵ_c on weekly incidence of COVID-19. Different curves correspond to different coefficients of variation (CV) of the degree distribution of the contact network.

Supplementary Note 9 Numerical evaluation of steady-state epidemic dynamics

Numerically evaluating the steady state of the SIS dynamics in Eq. (1) of the main textin the absence of prevention, i.e. $g_k = 0 \; \forall \; k$, is needed to compute z, ϕ, ψ. We did it with `DifferentialEquations.jl`[32] in Julia.[33] We used a cutoff maximum degree of 1000. Changing it to $10,000$ did not change the results.

To fix prevalence I (when needed), we numerically estimated the transmission probability, λ, that yielded the desired prevalence at the steady state. We used the optimization package `Optim.jl`.[34]

References

[1] Hansson, D., Strömdahl, S., Leung, K. Y. & Britton, T. Introducing pre-exposure prophylaxis to prevent hiv acquisition among men who have sex with men in sweden: insights from a mathematical pair formation model. *BMJ Open* **10**, e033852 (2020).

[2] Newman, M. *Networks* (Oxford University Press, Oxford, New York, 2018), second edn.

[3] Garnett, G. P. & Anderson, R. M. Factors controlling the spread of hiv in heterosexual communities in developing countries: patterns of mixing between different age and sexual activity classes. *Philosophical Transactions of the Royal Society of London. Series B, Biological Sciences* **342**, 137–159 (1993).

[4] Nichols, B. E., Boucher, C. A. B., van der Valk, M., Rijnders, B. J. A. & van de Vijver, D. A. M. C. Cost-effectiveness analysis of pre-exposure prophylaxis for HIV-1 prevention in the Netherlands: a mathematical modelling study. *Lancet Infect. Dis.* **16**, 1423–1429 (2016).

[5] Pastor-Satorras, R., Castellano, C., Van Mieghem, P. & Vespignani, A. Epidemic processes in complex networks. *Rev. Mod. Phys.* **87**, 925–979 (2015).

[6] Sherman, J. & Morrison, W. J. Adjustment of an inverse matrix corresponding to a change in one element of a given matrix. *Ann. Math. Stat.* **21**, 124–127 (1950).

[7] Weiss, K. M. *et al.* Egocentric sexual networks of men who have sex with men in the United States: Results from the ARTnet study. *Epidemics* **30**, 100386 (2020).

[8] Baggaley, R. F., White, R. G. & Boily, M.-C. HIV transmission risk through anal intercourse: systematic review, meta-analysis and implications for HIV prevention. *International Journal of Epidemiology* **39**, 1048–1063 (2010).

[9] UNAIDS. Key population atlas (2021). URL `https://aidsinfo.unaids.org/`.

[10] Piñeirúa, A. *et al.* The hiv care continuum in latin america: challenges and opportunities. *The Lancet Infectious Diseases* **15**, 833–839 (2015).

[11] Stannah, J. *et al.* Hiv testing and engagement with the hiv treatment cascade among men who have sex with men in africa: a systematic review and meta-analysis. *The lancet. HIV* **6**, e769–e787 (2019).

[12] Aghaizu, A. *et al.* Sexual behaviours, HIV testing, and the proportion of men at risk of transmitting and acquiring HIV in London, UK, 2000–13: a serial cross-sectional study. *Lancet HIV* **3**, e431–e440 (2016). URL `https://www.thelancet.com/journals/lanhiv/article/PIIS2352-3018(16)30037-6/fulltext`. Publisher: Elsevier.

[13] Marcus, U., Hickson, F., Weatherburn, P., Schmidt, A. J. & Network, E. Prevalence of hiv among msm in europe: comparison of self-reported diagnoses from a large scale internet survey and existing national estimates. *BMC public health* **12**, 978 (2012).

[14] Vourli, G. *et al.* Human immunodeficiency virus continuum of care in 11 european union countries at the end of 2016 overall and by key population: Have we made progress? *Clin. Infect. Dis.* **71**, 2905–2916 (2020).

[15] Coelho, L. E. *et al.* The prevalence of hiv among men who have sex with men (msm) and young msm in latin america and the caribbean: A systematic review. *AIDS Behav.* **25**, 3223–3237 (2021).

[16] Mason, K. *et al.* A cross-sectional analysis of population demographics, hiv knowledge and risk behaviors, and prevalence and associations of hiv among men who have sex with men in the gambia. *AIDS research and human retroviruses* **29**, 1547–1552 (2013).

[17] Baral, S. *et al.* Hiv prevalence, risks for hiv infection, and human rights among men who have sex with men (msm) in malawi, namibia, and botswana. *PloS One* **4**, e4997 (2009).

[18] Hladik, W. *et al.* Hiv infection among men who have sex with men in kampala, uganda–a respondent driven sampling survey. *PloS One* **7**, e38143 (2012).

[19] Hladik, W. *et al.* Men who have sex with men in kampala, uganda: Results from a bio-behavioral respondent driven sampling survey. *AIDS Behav.* **21**, 1478–1490 (2017).

[20] Holland, C. E. *et al.* Using population-size estimation and cross-sectional survey methods to evaluate hiv service coverage among key populations in burkina faso and togo **131**, 773–782 (2016).

[21] Hessou, P. H. S. *et al.* Comparison of the prevalence rates of hiv infection between men who have sex with men (msm) and men in the general population in sub-saharan africa: a systematic review and meta-analysis. *BMC public health* **19**, 1634 (2019).

[22] Djomand, G., Quaye, S. & Sullivan, P. S. Hiv epidemic among key populations in west africa **9**, 506–513 (2014).

[23] Sheehy, M. *et al.* High levels of bisexual behavior and factors associated with bisexual behavior among men having sex with men (msm) in nigeria. *AIDS care* **26**, 116–122 (2014).

[24] Wirtz, A. L. *et al.* Geographical disparities in hiv prevalence and care among men who have sex with men in malawi: results from a multisite cross-sectional survey. *The lancet. HIV* **4**, e260–e269 (2017).

[25] Park, J. N. *et al.* Hiv prevalence and factors associated with hiv infection among men who have sex with men in cameroon. *J. Int. AIDS Soc.* **16**, 18752 (2013).

[26] Sullivan, P. S. *et al.* Hiv prevalence and incidence in a cohort of south african men and transgender women who have sex with men: the sibanye methods for prevention packages programme (mp3) project. *J. Int. AIDS Soc.* **23 Suppl 6**, e25591 (2020).

[27] Zhang, Y. *et al.* Antiretroviral drug use and hiv drug resistance among msm and transgender women in sub-saharan africa. *AIDS (London, England)* **32**, 1301–1306 (2018).

[28] Baral, S. *et al.* A cross-sectional assessment of population demographics, hiv risks and human rights contexts among men who have sex with men in lesotho. *J. Int. AIDS Soc.* **14**, 36 (2011).

[29] Mossong, J. *et al.* Social contacts and mixing patterns relevant to the spread of infectious diseases. *PLoS medicine* **5**, e74 (2008).

[30] Endo, A., for the Mathematical Modelling of Infectious Diseases COVID-19 Working Group, C., Abbott, S., Kucharski, A. J. & Funk, S. Estimating the overdispersion in covid-19 transmission using outbreak sizes outside china. *Wellcome Open Research* **5**, 67 (2020).

[31] Pullano, G. *et al.* Underdetection of cases of covid-19 in france threatens epidemic control. *Nature* **590**, 134–139 (2021).

[32] Rackauckas, C. & Nie, Q. Differentialequations.jl–a performant and feature-rich ecosystem for solving differential equations in julia. *Journal of Open Research Software* **5** (2017).

[33] Bezanson, J., Karpinski, S., Shah, V. B. & Edelman, A. Julia: A fast dynamic language for technical computing. *arXiv preprint arXiv:1209.5145* (2012).

[34] Mogensen, P. K. & Riseth, A. N. Optim: A mathematical optimization package for Julia. *Journal of Open Source Software* **3**, 615 (2018).
