# Supplementary material for: Non-selective distribution of infectious disease prevention may outperform risk-based targeting
Source: Nat Commun. 2022 May 31;13:3028. doi: 10.1038/s41467-022-30639-3 (PMC9156732; doi:10.1038/s41467-022-30639-3)
Supplement: Supplementary file 3 — Reporting Summary [file 41467_2022_30639_MOESM3_ESM.pdf]

nature portfolio

Corresponding author(s): Eugenio Valdano

Last updated by author(s): Apr 7, 2022

Reporting Summary

Nature Portfolio wishes to improve the reproducibility of the work that we publish. This form provides structure for consistency and transparency in reporting. For further information on Nature Portfolio policies, see our Editorial Policies and the Editorial Policy Checklist.

Statistics

For all statistical analyses, confirm that the following items are present in the figure legend, table legend, main text, or Methods section.

n/a	Confirmed	
☐	☒	The exact sample size (n) for each experimental group/condition, given as a discrete number and unit of measurement
☒	☐	A statement on whether measurements were taken from distinct samples or whether the same sample was measured repeatedly
☒	☐	The statistical test(s) used AND whether they are one- or two-sided<br>*Only common tests should be described solely by name; describe more complex techniques in the Methods section.*
☐	☒	A description of all covariates tested
☒	☐	A description of any assumptions or corrections, such as tests of normality and adjustment for multiple comparisons
☒	☐	A full description of the statistical parameters including central tendency (e.g. means) or other basic estimates (e.g. regression coefficient) AND variation (e.g. standard deviation) or associated estimates of uncertainty (e.g. confidence intervals)
☒	☐	For null hypothesis testing, the test statistic (e.g. F, t, r) with confidence intervals, effect sizes, degrees of freedom and P value noted<br>*Give P values as exact values whenever suitable.*
☒	☐	For Bayesian analysis, information on the choice of priors and Markov chain Monte Carlo settings
☒	☐	For hierarchical and complex designs, identification of the appropriate level for tests and full reporting of outcomes
☒	☐	Estimates of effect sizes (e.g. Cohen's d, Pearson's r), indicating how they were calculated

Our web collection on statistics for biologists contains articles on many of the points above.

Software and code

Policy information about availability of computer code

Data collection	No data was collected in this study.
Data analysis	Code is in Julia (v 1.5.3). Libraries used are DifferentialEquations, DiffEqParamEstim, Optim. References to the documentation are provided in the Supplementary Information. The code is accessible here: https://github.com/steinegg/non_selective_distribution_prophylaxis and has the following DOI: https://doi.org/10.5281/zenodo.6418584

For manuscripts utilizing custom algorithms or software that are central to the research but not yet described in published literature, software must be made available to editors and reviewers. We strongly encourage code deposition in a community repository (e.g. GitHub). See the Nature Portfolio guidelines for submitting code & software for further information.

Data

Policy information about availability of data

All manuscripts must include a data availability statement. This statement should provide the following information, where applicable:

- Accession codes, unique identifiers, or web links for publicly available datasets
- A description of any restrictions on data availability
- For clinical datasets or third party data, please ensure that the statement adheres to our policy

Estimates of HIV prevalence and treatment coverage as discussed in Supplementary Note 5 and Supplementary note 6 are available from the cited references in the Supplementary Information, and from UNAIDS at https://aidsinfo.unaids.org/ (accessed February 2022).

Field-specific reporting

Please select the one below that is the best fit for your research. If you are not sure, read the appropriate sections before making your selection.

☒ Life sciences ☐ Behavioural & social sciences ☐ Ecological, evolutionary & environmental sciences

For a reference copy of the document with all sections, see nature.com/documents/nr-reporting-summary-flat.pdf

Life sciences study design

All studies must disclose on these points even when the disclosure is negative.

Sample size	We used community-level estimates on HIV prevalence, antiretroviral treatment coverage, viral suppression rate, and PrEP effectiveness, in communities of men-having-sex-with-men. All estimates come from previously published studies, and reference are provided within the main text, and Supplementary Information. Sample sizes to produce these community-level estimates were determined, and shown to be sufficiently large, in the studies that made these estimates available, and that we cite. The analyses that we performed were conducted at aggregate levels, using the aforementioned population-level estimates. As such, they did not require the identification of any sample.
Data exclusions	We excluded communities of men-having-sex-with-men for which estimates of HIV prevalence, antiretroviral treatment coverage, viral suppression rate, were not available in the literature.
Replication	The steps of the analytical derivation are thoroughly described in the text, and the Reader can replicate them. The data used are published estimates, publicly available. The numerical part of our study is described and can be replicated by the Reader both by implementing their own code, and by using ours, which we provide.
Randomization	We utilized already collected databases, so randomization was not possible, or relevant. All analyses were conducted at aggregate levels (nothing was done at the individual-level).
Blinding	Blinding is not relevant because we carried out a secondary analysis using non-identifiable data.

Reporting for specific materials, systems and methods

We require information from authors about some types of materials, experimental systems and methods used in many studies. Here, indicate whether each material, system or method listed is relevant to your study. If you are not sure if a list item applies to your research, read the appropriate section before selecting a response.

Materials & experimental systems

n/a	Involved in the study	
☒	☐	Antibodies
☒	☐	Eukaryotic cell lines
☒	☐	Palaeontology and archaeology
☒	☐	Animals and other organisms
☒	☐	Human research participants
☒	☐	Clinical data
☒	☐	Dual use research of concern

Methods

n/a	Involved in the study	
☒	☐	ChIP-seq
☒	☐	Flow cytometry
☒	☐	MRI-based neuroimaging
